# Implementation of biological variation-based analytical performance specifications in the laboratory: Stringent evaluation of Improvacutor blood collection tubes

**DOI:** 10.1371/journal.pone.0189882

**Published:** 2017-12-20

**Authors:** Hee-Jung Chung, Yoon Kyung Song, Sung Kuk Hong, Sang-Hyun Hwang, Hee Seung Seo, Dong Hee Whang, Myung-Hyun Nam, Do Hoon Lee

**Affiliations:** 1 Department of Laboratory Medicine, National Cancer Center, Goyang, South Korea; 2 Department of Laboratory Medicine, Asan Medical Center, University of Ulsan College of Medicine, Seoul, Korea; 3 Department of Laboratory Medicine, Seoul Paik Hospital, Inje University College of Medicine, Seoul, Korea; 4 Department of Laboratory Medicine, Korea University College of Medicine, Korea University Ansan Hospital, Ansan, Korea; Holbæk Hospital, DENMARK

## Abstract

Recently, because the quality of laboratory analyses has increased along with the need for quality improvement, several external quality control bodies have adapted performance specifications using the Desirable Biological Variation Database, termed “Ricos goals”; these criteria are more stringent than those presented in CLIA 88. In this study, we aimed to validate newly introduced serum separator tubes, Improvacutor, for routine clinical chemistry testing in accordance with Ricos goals and CLIA 88. Blood samples were collected from 100 volunteers into three types of serum vacuum tubes: Greiner Vacuette, Becton Dickinson (BD) Vacutainer, and Improve Improvacutor. The samples were subjected to 16 routine chemistry tests using a TBA-200fr NEO chemistry autoanalyzer. In the comparison analysis, all 16 test results were acceptable according to CLIA 88. However, in the comparison of Improve and BD tubes, creatinine showed 4.31% (+0.08 μmol/L) bias. This slightly exceeded the Desirable Specification for Inaccuracy Ricos limit of ±3.96%, but still satisfied the CLIS88 limit of ±26.52 μmol/L. The remaining 15 analytes performed acceptably according to the Desirable Specifications of Ricos. The correlation coefficient of 12 analytes was greater than 0.95 in Passing-Bablok regression analysis among the three tubes, but was lower for four analytes: calcium, sodium, potassium, and chloride. In the stability assay, only potassium tested in the Greiner tube revealed a larger positive bias (2.18%) than the Ricos Desirable Specification for Inaccuracy based on biologic variation (1.8%). The BD tube also showed a positive bias of 1.74%, whereas the new Improve tube showed the smallest positive bias of 1.17% in potassium level after 72 h storage. Thus, the results of this study demonstrate that recently introduced analytical performance specifications based on components of biological variation (Rico's goal) could be extended to criterion for performance evaluation and applied.

## Introduction

The Clinical Laboratory Improvement Amendments of 1988 (CLIA 88) constitute the United States federal regulatory standards that apply to all clinical laboratory testing performed in humans, except for clinical trials and basic research [1]. Although CLIA 88 are not sufficiently strict for laboratories to maintain high quality standards, CLIA requirements nevertheless are widely used as standards for analytical performance because no other specific and reliable criteria are available.

When repeated measurements are made over time in an individual, even under standardized conditions there is considerable variability in the test results. This phenomenon, termed biological variation, makes it difficult to determine whether there is a difference between test results [2]. Biological variation may also be used to establish guidelines with respect to bias, coefficient of variation (CV), and total allowable error [3, 4]. Notably, Dr. Carmen Ricos and colleagues have established Desirable Specifications for imprecision, inaccuracy, and total allowable error, calculated from data on within-subject and between-subject biologic variation [3]. This established criteria based on an extensive database are called “Ricos goals” and are updated by the European Federation of Clinical Chemistry and Laboratory Medicine (EFLM) since 2014. In February of 2017, the EFLM Task and Finish Group on Allocation of Laboratory Tests to Different Models for Performance Specifications (TFG-DM) introduced analytical performance specifications based on components of biological variation as a model for analytical performance specifications in the first EFLM Strategic Conference [5]. Many external quality control (EQA) bodies have recently established their own EQA acceptance limits of quality, which include some limits described as Ricos goals, such as French EQA ProBioQual and Spanish EQA providers [6–8]. Other than EQA acceptance limits, biological variation-based specifications for several analytes are sometimes used as quality requirements [9–13]. The level of performance of the instrument and the technician is greatly vary from laboratory to laboratory. Therefore, these quality requirements using Ricos goal can be relatively strict and high in applying as a criteria for evaluating analytical performance.

The major cause of pre-analytical error of clinical laboratory tests is blood sampling, handling, and disposal. Currently, evacuated blood collection tubes are widely used to collect and store blood, guaranteeing basic conditions for accurate analysis [14]. Evacuated blood collection tubes automatically draw a predetermined blood volume [15]. Commonly used evacuated blood collection tubes in clinical laboratories are quite similar, but vary in terms of materials and additives, which can potentially affect test performance [16]. In Korea, two major evacuated tube products dominate the market: Vacuette (Greiner Bio-One, Kremsmünster, Austria) and Vacutainer (Becton Dickinson, Franklin Lakes, NJ, USA). Because blood collection tubes function properly in most situations, most laboratories are unaware of their complexity and limitations [17, 18].

The purpose of this study was to validate the newly introduced plastic serum separator tubes (SSTs; Improvacutor [Improve Medical, Guangzhou, China]) for routine clinical chemistry testing in according to the Ricos goals and CLIA 88.

## Materials and methods

### Subjects

This study was conducted between August and September 2015 at the National Cancer Center, South Korea, a 600-bed tertiary care hospital. The Department of Laboratory Medicine conducts 62.7 million annual tests and participates in international and nation-wide EQA programs. A total of 100 patients were included in this study, based on the Clinical and Laboratory Standards Institute (CLSI) EP09-A3 guideline [19]. Patients who visited the outpatient clinic for cancer treatment follow-up or who visited the Health Promotion Center were included. The patients consisted of 26 men and 74 women ranging from 21 to 70 years of age (median age: 42 years). The protocol was approved by the National Cancer Center Institutional Review Board and patients provided written informed consent before enrollment.

### Sample collection

Venous blood was collected by routine phlebotomy by two expert phlebotomists in accordance with the CLSI GP41-Ed7 guideline [20]. The three types of tubes evaluated in this study were as follows: Tube I, Vacuette SST II Advance (Greiner Bio-One; lot number: 1503008); Tube II, Vacutainer SST (BD, Vacutainer; lot number: 5320998); Tube III, Improvacutor SST (Improve Medical; lot number: C65005). To reduce the cross-scan error, blood was collected in the order of Tube II—Tube I—Tube III, Tube I—Tube III—Tube II, and Tube III—Tube II—Tube I, alternately. Exclusion criteria were as follows: underfilled tubes, hemolyzed samples according to hemolytic index (above 20), and samples with delayed analysis by more than 2 h after phlebotomy. No samples met the exclusion criteria. Blood was collected by venipuncture with a 20-G straight needle (BD) directly into three serum vacuum tubes with a clot activator and gel separator. The three tubes were gently inverted to mix the clot activator evenly with the blood. All the sample tubes were left in the upright position for 30 min at room temperature (23°C) to allow for complete clotting before centrifugation. After clot formation, all tubes were centrifuged together at 3000 rpm (1811 × *g*) for 10 min at room temperature in a swing bucket centrifuge (Labmaster ABC-CB200r; Hanlab, Anyang-si, South Korea). These centrifugation conditions were consistent with the CLSI GP44-A4 guideline [21].

### Comparison and stability analysis

All sample tubes were evaluated in 16 routine chemistry tests using a TBA-200FR NEO chemistry autoanalyzer (Toshiba Medical Systems, Tokyo, Japan), according to the manufacturer’s specifications with the recommended reagents. These tests included the following analytes: total calcium (Ca), phosphorus (PHOS), glucose (GLU), blood urea nitrogen (BUN), uric acid (UA), cholesterol (CHOL), total protein (TP), albumin (ALB), total bilirubin (TB), alkaline phosphatase (ALP), aspartate aminotransferase (AST), alanine aminotransferase (ALT), creatinine (CREA), sodium (Na), potassium (K), and chloride (Cl). All tests were conducted under the same conditions as routine chemistry analysis. Calibration and quality control were performed daily.

For comparison study, all tubes were tested simultaneously immediately after centrifugation. The stability test was performed mimicking the same re-test process used in a clinical laboratory to examine the changes owing to storage. The initial results from a fresh sample for each tube were compared with results from samples preserved for 72 h at 4°C. The 72-h stability assay was conducted by wrapping the tubes after completing the initial tests.

### Statistical analysis

Statistical analysis was performed using Microsoft Excel with R program 3.3.2 under CentOS Linux 7. In comparison tests of the three types of tubes, differences among the results were evaluated by analysis of variance (ANOVA) with post-hoc tests as needed. In stability tests, differences between the results from 0 and 72 h were evaluated by Student’s paired *t*-tests. The level of significance for all statistical analyses was set to *P* < 0.05. Data are expressed as the means ± standard deviation (SD). Bland-Altman plots were constructed for the 16 analytes in the comparative analysis [22]. Differences in test results between the tubes were compared in terms of the CLIA 88 regulation and Ricos goals [3, 4].

## Results

### Comparative analysis of the three types of SSTs

**Fig 1:**
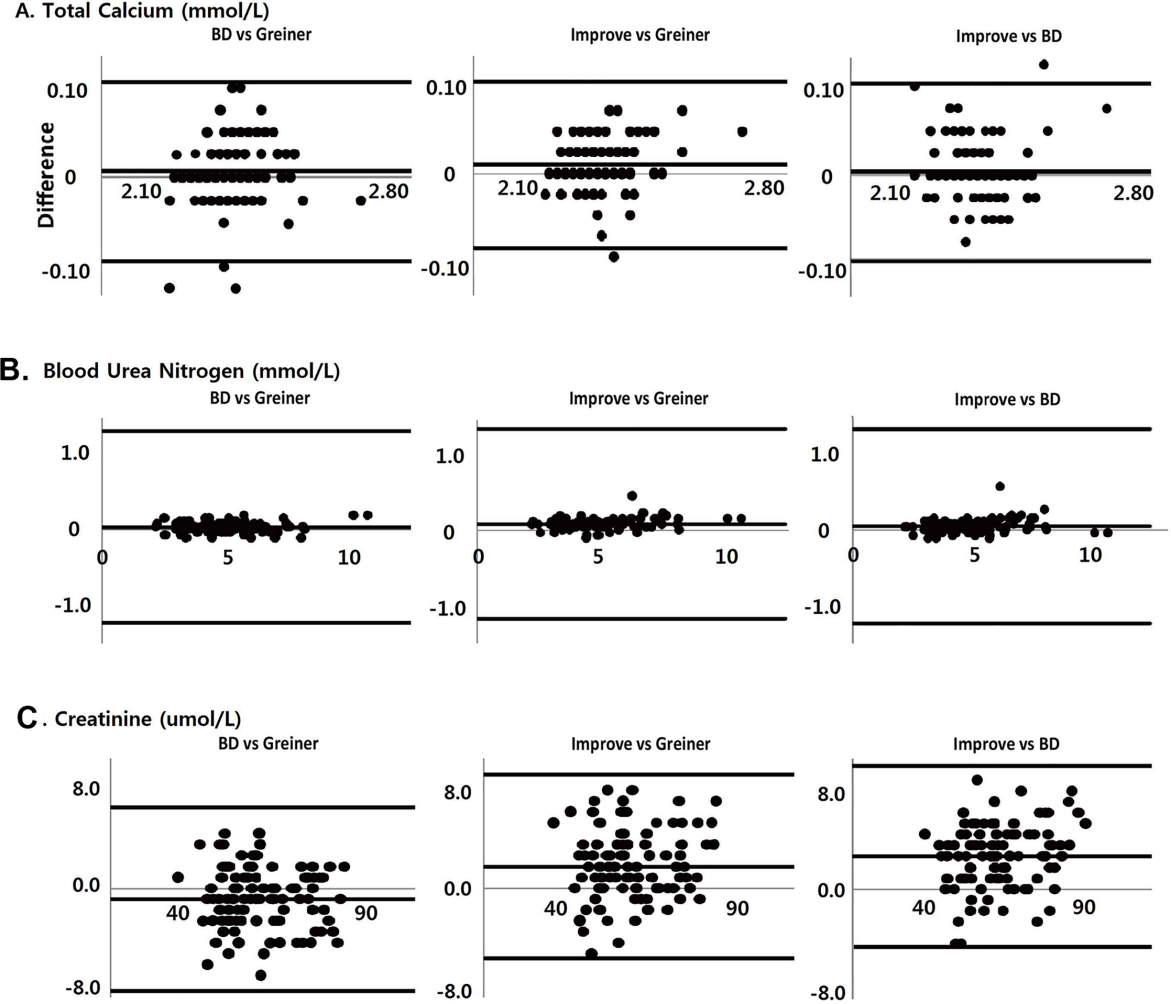
Bland-Altman plots for the representative 3 routine chemistry analytes: (A) Ca,(B) BUN, and (C) CREA for the three types of tubes. To compare the absolute differences vertically, three figures were generated for each analyte (BD Vacutainer versus Greiner Vacuette, Improvacutor versus Greiner Vacuette, and Improve Improvacutor versus BD Vacutainer) from the left. Solid lines denote the average difference and ± 2SD in SI units.

The results of comparisons of Greiner Vacuette, BD Vacutainer, and Improve Improvacutor tubes are shown in Table 1. In ANOVA, all 16 analytes showed no significant difference among the three types of tubes. Thus, the subsequent post-hoc test results were omitted because there were no significant differences among the three types of tubes. Fig 1 shows absolute differences in the representative 3 analytes; Ca, BUN, and CREA. To compare the absolute differences vertically, three types of comparison figures are shown together for each analyte. The correlation coefficient of 12 analytes was greater than 0.95 in Passing-Bablok regression analysis among the three tubes but lower for four analytes; Ca, Na, K, and Cl. Graphical differences are shown in Fig 1.

**Table 1:** Statistical analysis of the results of blood samples from three types of collection tubes (mean ± SD).

Test (unit)		BD Vacutainer	Greiner Vacuette	Improve Improvacutor	*P* value in ANOVA
Ca	(mmol/L)	2.38 ± 0.10	2.38 ± 0.10	2.39 ± 0.10	0.753
PHOS	(mmol/L)	1.21 ± 0.16	1.21 ± 0.16	1.21 ± 0.16	0.964
GLU	(mmol/L)	5.8 ± 2.2	5.8 ± 2.2	5.9 ± 2.3	0.994
BUN	(mmol/L)	5.0 ± 1.6	5.0 ± 1.6	5.1 ± 1.7	0.948
UA	(mmol/L)	0.28 ± 0.08	0.28 ± 0.07	0.28 ± 0.08	0.994
CHOL	(mmol/L)	5.1 ± 1.0	5.1 ± 1.0	5.1 ± 1.0	0.916
TP	(g/L)	76.2 ± 3.8	75.8 ± 3.8	76.4 ± 4.0	0.553
ALB	(g/L)	44.1 ± 2.4	43.9 ± 2.3	44.2 ± 2.4	0.596
TB	(μmol/L)	11.6 ± 5.3	11.5 ± 5.3	11.6 ± 5.3	0.983
ALP	(IU/L)	67.0 ± 22.2	66.4 ± 21.8	67.4 ± 22.3	0.954
AST	(IU/L)	21.9 ± 18.7	21.9 ± 18.6	21.9 ± 18.8	1.000
ALT	(IU/L)	20.3 ± 17.3	20.4 ± 17.4	20.4 ± 17.5	0.999
CREA	(μmol/L)	61.9 ± 11.5	62.8 ± 12.4	64.5 ± 12.4	0.284
Na	(mmol/L)	141.4 ± 1.7	141.3 ± 1.7	141.6 ± 1.6	0.597
K	(mmol/L)	4.4 ± 0.4	4.4 ± 0.4	4.4 ± 0.4	0.807
Cl	(mmol/L)	104.0 ± 2.0	103.8 ± 2.3	103.9 ± 2.0	0.780

*P* values were calculated by ANOVA and post-hoc tests. The decimal point was marked as the reporting unit of clinical results. For example, the mean of calcium is shown as 2.38 mmol/L because the calcium level is clinically reported as 3.0 mmol/L.

The test results were compared with the tolerance of CLIA 88 and Desirable Specifications of Ricos, as shown in Table 2. According to CLIA 88, all 16 test results were acceptable. However, according to Ricos goals, CREA showed 4.31% (+0.08 μmol/L) bias upon comparison between Improve and BD tubes. This slightly exceeded the Desirable Specification for Inaccuracy Ricos limit of ± 3.96% but still satisfied the CLIS88 limit of ± 26.52 μmol/L. The remaining test results were acceptable according to the Desirable Specifications of Ricos. Laboratory Imprecision results for these analytes, which represent the repeatability of a laboratory, are also described in Table 2.

**Table 2:** Bias analysis and comparison of the results of blood sample analysis from the three types of collection tubes according to desirable specifications of Ricos and CLIA 88.

Analyte	Laboratory Imprecision^a^, CV (%)	Bias (%)^b^			Desirable Specification for Inaccuracy ^c^ (%)	CLIA 88	
		BD vs Greiner	Improve vs Greiner	Improve vs BD			
Ca	0.98	0.28	0.44	0.16	0.82	Target ±	0.25 mmol/L
PHOS	1.37	0.43	0.45	0.03	3.4	Target ±	10% ^d^
GLU	0.72	-0.16	0.43	0.59	2.3	Target ±	0.333 mmol/L
BUN	1.25	0.61	1.51	0.90	5.6	Target ±	0.714 mmol/L
UA	1.04	-0.06	0.32	0.38	4.9	Target ±	17%
CHOL	0.76	0.58	1.17	0.59	4.1	Target ±	10%
TP	0.78	0.49	0.78	0.29	1.4	Target ±	10%
ALB	0.74	0.41	0.77	0.36	1.4	Target ±	10%
TB	1.47	0.74	1.19	0.44	8.95	Target ±	20%
ALP	1.41	0.83	1.43	0.60	6.7	Target ±	30%
AST	1.40	0.05	-0.18	-0.23	6.5	Target ±	20%
ALT	2.44	-0.59	-0.20	0.39	11.48	Target ±	20%
CREA	1.35	-1.40	2.85	4.31	3.96	Target ±	26.52 μmol/L
Na	0.47	0.08	0.17	0.09	0.23	Target ±	4.0 mmol/L
K	0.81	0.80	0.73	-0.07	1.8	Target ±	0.5 mmol/L
Cl	0.50	0.18	0.16	-0.02	0.5	Target ±	5%

^a^ Laboratory imprecision was verified in accordance with CLSI document EP15-A3 [23]

^b^ Bias (%) = ([test tube mean − reference tube mean] / reference tube mean) × 100. The reference tube was the Greiner tube. In comparisons of the Improve versus BD tubes, the newly introduced Improve tubes were considered as the test tubes.

^c^ Ricos goal, which is the desirable bias derived from biological variation [3, 4]. Decimal point was not unified and was marked as shown in the database. By rounding the decimal point and unifying it to one decimal place, it is possible to cause an error in judgment of acceptability.

^d^ Allowable total error limits for linearity in the CAP survey were adapted because there is no criterion in CLIA 88.

### Stability assay for the three types of SSTs

Table 3 shows 72-h stability assay results for Greiner Vacuette, BD Vacutainer, and Improve Improvacutor tubes for 16 routine chemistry analytes. There were 9 test items with statistically significant differences for BD Vacutainer tubes, 11 for Greiner Vacuette tubes, and 9 for Improve Improvacutor tubes. A total of five analytes (PHOS, TB, ALP, ALT, and K) showed significant bias in the same manner in all three tubes. A total of four analytes (GLU, BUN, UA, and CHOL) showed significant bias in the same manner in both BD and Greiner tubes but not in Improve tubes. Improve tubes showed significant bias after 72-h storage for analytes Ca, AST, CREA, and Cl as shown in Table 3.

**Table 3:** Comparison of test results before and after 72-h storage of the same samples at 4°C.

Analyte (unit)		BD		Greiner		Improve		Desirable specification for inaccuracy ^b^ (%)
		Bias (%)^a^	*P* value	Bias (%)^a^	*P* value	Bias (%)^a^	*P* value	
Ca	(mmol/L)	−0.3	0.10	0.1	0.72	−0.5	**<** *0*.*01*	0.82
PHOS	(mmol/L)	2.1	< *0*.*01*	2.2	< *0*.*01*	1.4	< *0*.*01*	3.4
GLU	(mmol/L)	0.3	*0*.*03*	0.6	< *0*.*01*	0.1	0.43	2.3
BUN	(mmol/L)	0.8	< *0*.*01*	1.2	< *0*.*01*	0.4	0.09	5.6
UA	(mmol/L)	0.6	*0*.*01*	0.5	*0*.*01*	0.4	0.06	4.9
CHOL	(mmol/L)	0.4	< *0*.*01*	0.7	< *0*.*01*	0.2	0.08	4.1
TP	(g/L)	−0.1	0.57	0.4	< *0*.*01*	0.0	0.8	1.4
ALB	(g/L)	0.1	0.41	0.3	*0*.*02*	0.2	0.23	1.4
TB	(μmol/L)	−5.9	< *0*.*01*	−3.3	< *0*.*01*	−4.1	< *0*.*01*	8.95
ALP	(IU/L)	−0.5	*0*.*01*	-0.6	< *0*.*01*	−1.5	< *0*.*01*	6.7
AST	(IU/L)	0.6	0.06	0.6	0.12	−1.0	< *0*.*01*	6.5
ALT	(IU/L)	−2.2	< *0*.*01*	−2.4	< *0*.*01*	−2.9	< *0*.*01*	11.48
CREA	(μmol/L)	−0.2	0.64	−0.3	0.59	1.6	< *0*.*01*	3.96
Na	(mmol/L)	0.0	0.79	0.1	0.25	−0.1	0.14	0.23
K	(mmol/L)	1.7	< *0*.*01*	2.2	< *0*.*01*	1.2	< *0*.*01*	1.8
Cl	(mmol/L)	−0.1	0.33	0.2	0.21	−0.1	*0*.*03*	0.5

*P* values were calculated by Student’s paired *t* -test.

Statistically significant values are shown in italic.

^a^ Bias (%) = ([72 h tube mean − 0 h tube mean] / 0 h tube mean) × 100, in each tube.

^b^ Ricos goal, which is the desirable bias derived from biological variation [3, 4]. Decimal point was not unified and was marked as shown in the database. By rounding the decimal point and unifying it to one decimal place, it is possible to cause an error in judgment of acceptability.

When the stability assay results were compared with the clinical tolerance of CLIA 88, all 16 test results were acceptable. In comparison, a total of 15 test results were acceptable according to Ricos goals, with the exception of potassium. In Greiner tubes, K revealed a larger positive bias (2.18%) than the Desirable Specification for Inaccuracy based on biologic variation (1.8%). BD tubes also showed a positive bias of 1.74%, whereas the new Improve tube showed the smallest positive bias of 1.17% in K level after 72 h of storage.

Statistically, Improve tubes showed similar stability as BD tubes and better than that of Greiner tubes. Clinically, however, only K in Greiner tubes showed meaningful increase. The biases in the remainder were within the range of expectable biological difference according to Rico’s database, in all three tubes.

## Discussion

Although CLIA 88 regulatory standards for US clinical laboratories are not sufficiently strict, they have been the most widely used criteria for acceptable analytical performance because there are no other specific and reliable criteria available [1] owing to the necessity for analysis of many analytes. Biological variation may also be used to establish guidelines on bias, CV, and total allowable error [8, 10–12]. As interest in improving the quality of clinical laboratories is increasing [6, 7, 24], CLIA 88 is now considered to be unsuitable as a performance criterion in high-quality laboratories that pursue high and strict standards. However, although biological variation was considered during the selection of quality requirements, for several analytes, such as ALT, a biological variation-based quality requirement is too stringent given the analytical performance that is currently possible for the majority of diagnostic instruments. Moreover, in order to slightly improve the quality of the laboratory, substantially more quality-related expenses are required. For example, internal quality control is performed at a higher frequency; expenses are required to participate in external quality control; for analytes without an EQA program, regular interlaboratory comparison is needed; and for delta-checked samples, labor and time are required to identify the cause, and sometimes re-testing and/or re sampling is needed to exclude specimen misidentification or mislabeling. Therefore, the quality of clinical tests and the cost of testing are inversely related. Thus, as the need for clinical laboratories to improve accuracy and precision is increased, the quality-related expense is also increased [6, 24].

Even though it incurs relatively high expenses, an accuracy-based surveillance program that involves comparison with the “true value” rather than the peer-group mean has recently been initiated. Our laboratory is located at a 600-bed cancer center, and routine chemistry tests are performed for international and nationwide external quality control programs including programs administered by the College of American Pathologists survey. Laboratory precision for 16 routine chemistry tests evaluated in this study revealed a low CV, indicating a good internal quality control, as shown in Table 2, even including a low level of total bilirubin (TB), which usually shows a high CV%. In this study, we concluded that there were no significant differences among the three types of tubes, even according to biological variation-based quality requirements using criteria that were stricter than CLIA 88. Furthermore, these three types of tubes were able to fulfill the strict goals of the Desirable Specification of Ricos. This may have been possible because the quality of our laboratory has been maintained sufficiently for more than 30 years with notable effort and expense taken for quality control.

Separator gels are used to separate serum from clotted whole blood or plasma from cells. Separator gels are typically made of viscous liquids, fillers, or tackifiers with substances like dibenzylidene sorbitol as a gelling agent [25]. The inner surface of such tubes may have a hydrophobic coating to ensure adhesion of the separator gel and a complete barrier to prevent mixing between red blood cells and serum or plasma [26, 27]. Plastic tubes require clot activators that use either intrinsic or extrinsic pathways to ensure rapid and dense clot formation [26]. Clot activators can be added into tubes as small beads or paper-coated discs, or they can be sprayed on interior tube surfaces with a carrier (e.g., polyvinyl chloride, carboxymethyl cellulose, polyvinyl alcohol, or polyethylene oxide) [25, 26]. These carriers enable rapid dissolving of a clot activator in the blood so that the carriers diffuse into both serum and clots as the clotting is initiated [25]. Any new or modified blood collection product should ideally be thoroughly evaluated for any potential problems inherently caused during the downstream processing and analysis of samples [28]. When improperly used or because of problems related to their manufacturing, blood collection tubes can interfere with test results and thus adversely affect patient outcomes, decrease laboratory efficiency, delay test results, and increase the cost per test because of recollection and retesting [28]. When laboratory technicians change the brand of tubes they use, they should also perform a comparative tube evaluation [29]. The Bland-Altman plot effectively shows results of comparison between tubes [30]. The x-axis shows the mean of the results of the two methods ([A + B] / 2), whereas the y-axis represents the absolute difference between the two methods ([B − A]). In our National Cancer Center clinical laboratory, clinically reported results and instrumental raw results involve the same number of significant digits. For example, even though the test result on Na is 135.35, it is already treated as 135.4 by the measurement instrument. Thus, when the results show a miniscule difference, the difference is indicated similar to a semi-quantitative result in the figure.

A total of 16 routine chemistry analytes, except CREA in the comparison of Improve and BD tubes, were clinically acceptable according to Ricos goals. Improve Improvacutor tubes yielded satisfactory results compared with Greiner Vacuette and BD Vacutainer tubes. Additionally, the stability assay results were analyzed in accordance with the tolerance of CLIA 88 and Desirable Specification of Ricos. All 16 test results of stability were acceptable according to both standards.

In summary, the newly introduced Improve Improvacutor SSTs yielded generally satisfactory results compared with Greiner Vacuette and BD Vacutainer tubes. However, it is insufficient to evaluate the performance of all clinical laboratories against a broad standard such as CLIA88, as there is a need to differentiate laboratory performance criteria by applying much narrower and more stringent standards. Notably, tube performance was also acceptable when evaluated by means of the Ricos biological variation-based quality requirements. We therefore concluded that the tubes were acceptable for routine clinical chemistry laboratory tests because there were minimal differences among the test results from the three types of tubes and because the quality of results from our laboratory was sufficient to meet high stringency standards.

## Supporting information

S1 Table Correlation coefficient values between tubes in 16 analytes. Samples were drawn from the same patient into 3 tubes simultaneously and analyzed under the same conditions.
(DOCX)
