# Supplementary material for: Implementation of biological variation-based analytical performance specifications in the laboratory: Stringent evaluation of Improvacutor blood collection tubes
Source: PLoS One. 2017 Dec 20;12(12):e0189882. doi: 10.1371/journal.pone.0189882 (PMC5738124; doi:10.1371/journal.pone.0189882)
Supplement: S1 Table — Samples were drawn from the same patient into 3 tubes simultaneously and analyzed under the same conditions. (DOCX) [file pone.0189882.s001.docx]

**S1 Table.** **Correlation Coefficients value between tubes in 16 analytes. S**amples were thrawn from same patient into 3 tubes simultaneously and analyzed in the same condition.

|  | BD vs Greiner | Improvacutor vs Greiner | Improvacutor vs BD |
| --- | --- | --- | --- |
| Ca | 0.932 | 0.952 | 0.941 |
| PHOS | 0.987 | 0.992 | 0.992 |
| GLU | 0.998 | 0.999 | 0.999 |
| BUN | 0.999 | 0.999 | 0.999 |
| UA | 0.999 | 0.999 | 0.999 |
| CHOL | 0.997 | 0.997 | 0.996 |
| TP | 0.958 | 0.949 | 0.954 |
| ALB | 0.954 | 0.956 | 0.960 |
| TB | 0.996 | 0.994 | 0.995 |
| ALP | 0.999 | 0.998 | 0.999 |
| AST | 0.999 | 0.999 | 0.999 |
| ALT | 0.999 | 0.999 | 0.998 |
| CREA | 0.979 | 0.973 | 0.978 |
| Na | 0.903 | 0.913 | 0.922 |
| K | 0.886 | 0.850 | 0.848 |
| Cl | 0.781 | 0.788 | 0.955 |
